# Cerebrospinal fluid PCR: A new approach for the diagnosis of CNS sporotrichosis

**DOI:** 10.1371/journal.pntd.0008196

**Published:** 2020-07-16

**Authors:** Manoel Marques Evangelista Oliveira, Mauro de Medeiros Muniz, Rodrigo Almeida-Paes, Rosely Maria Zancope-Oliveira, Andrea D’Avila Freitas, Marco A. Lima, Maria Clara Gutierrez-Galhardo, Dayvison Francis Saraiva Freitas

**Affiliations:** 1 Laboratório de Taxonomia, Bioquímica e Bioprospecção de Fungos, Instituto Oswaldo Cruz, Fundação Oswaldo Cruz, Rio de Janeiro, Brazil; 2 Laboratório de Micologia, Instituto Nacional de Infectologia Evandro Chagas, Fundação Oswaldo Cruz, Rio de Janeiro, Brazil; 3 Serviço Médico, Instituto Nacional de Infectologia Evandro Chagas, Fundação Oswaldo Cruz, Rio de Janeiro, Brazil; 4 Laboratório de Pesquisa Clínica em Neuroinfecções, Instituto Nacional de Infectologia Evandro Chagas, Fundação Oswaldo Cruz, Rio de Janeiro, Brazil; 5 Laboratório de Pesquisa Clínica em Dermatologia Infecciosa, Instituto Nacional de Infectologia Evandro Chagas, Fundação Oswaldo Cruz, Rio de Janeiro, Brazil; University of Tennessee, UNITED STATES

## Introduction

Cases of meningoencephalitis have increased in the zoonotic hyperendemia of sporotrichosis in the state of Rio de Janeiro, Brazil [1–2]. The gold standard for the diagnosis of sporotrichosis is the isolation of *Sporothrix* spp. from clinical specimens [3], not always possible from the cerebrospinal fluid (CSF) of these patients, since fungus recovery in this specimen is difficult in most cases [4]. This limitation led us to pursue a new approach on central nervous system (CNS) sporotrichosis diagnosis based on existing molecular methodologies for the detection of *Sporothrix* spp. in skin samples [5–9]. Kano and colleagues (2003) designed species-specific primers for polymerase chain reaction (PCR) based on *Sporothrix schenckii sensu lato* chitin synthase 1 (*CHS1*) gene sequence and applied it in skin biopsy paraffin block [5]. Hu and colleagues (2003) used a nested PCR in human clinical samples and samples from infected mice, with the 18S rRNA gene sequence as target [6]. The assay was successfully used to detect *S*. *schenckii* DNA from strains from different areas of the world [7]. However, Mendoza and colleagues [8] compared the previously described nested PCR with conventional diagnostic methods, and the molecular methodology presented lower efficacy. Liu and colleagues [9], using the primer pair S2-R2 targeting the *CHS1* gene in the PCR of biopsy tissue, verified positive results in 25 out of 30 cases (83.3%). The nested PCR targeting the partial sequence of the 18S rRNA gene was the best choice in terms of sensitivity due to a low fungal burden in CSF [6–7].

Since the beginning of the hyperendemic sporotrichosis in 1998, patients with disseminated sporotrichosis followed up at the Instituto Nacional de Infectologia Evandro Chagas (INI), Fundação Oswaldo Cruz (Fiocruz), undergo a protocol with lumbar puncture because of the possible neurotropism of *S*. *brasiliensis*, the main involved species in this region. Thereby, our main purpose was to apply the nested PCR assay proposed by Hu and colleagues [6], slightly modified, for the diagnosis of CNS sporotrichosis.

## Methods

### Study site and samples

INI-Fiocruz is a national reference center for infectious diseases, located in Rio de Janeiro, Brazil. Samples of CSF from 5 patients with advanced AIDS and sporotrichosis, collected during a routine clinical investigation, were used in the analyses.

### Ethical aspects

All patients were included in a cohort of a study approved by the institutional Research Ethics Committee of the INI-Fiocruz, Brazil, approval number 3.095.183, and the data were analyzed anonymously.

### DNA extraction from clinical samples

Two hundred microliters of the CSF were used for DNA extraction using the QIAamp DNA mini kit (QIAGEN, Hilden, Germany), following all the manufacturer’s instructions. NanoDrop (Thermo Fisher Scientific, Waltham, Massachusetts, United States of America) was used to analyze the DNA concentration.

### DNA extraction control in reaction

**Fig 1:**
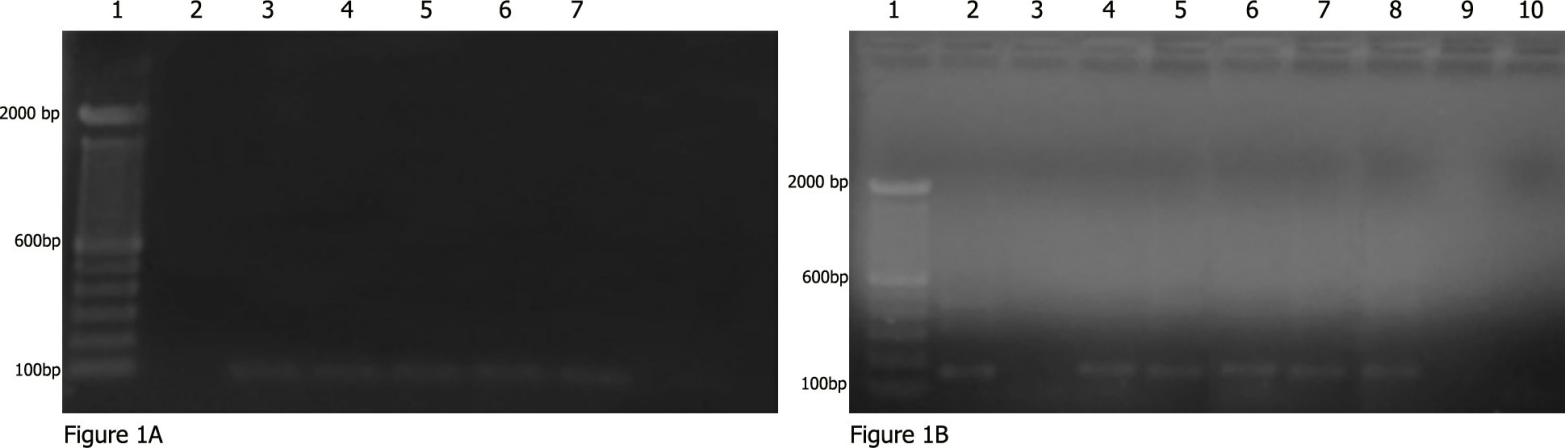
PCR amplification products in agarose gel electrophoresis **(A)** Detection of human β-globin DNA in all 5 patients (3 to 7) by the control PCR. (1) Molecular marker DNA ladder, 100 bp (Invitrogen), (2) negative control (Blank), and (3–7) DNA of the patient’s CSF. **(B)** Detection of *Sporothrix* by nested PCR obtained for the DNA of the patient’s CSF. (1) Molecular marker DNA ladder, 100 bp (Invitrogen), (2) positive control (DNA of *Sporothrix* spp.), (3) negative control (Blank), (4–8) DNA of the patients CSF, (9) negative control (DNA *Cryptococcus neoformans*), and (10) negative control (Blank). CSF, cerebrospinal fluid.

To validate the quality control for DNA extraction, the human β-globin gene was amplified in a separate PCR using the primers β-glob F (5’-GCAAGAAAGTGCTCGGTGC-3’) and β-glob R (5’-CACTCAGTGTGGCAAAGGTG-3’), according to a previous protocol (Fig 1A) [10]. *S*. *brasiliensis* DNA extracted from CBS120339 (former IPEC16490) strain was used in every batch of PCR as a positive control (Fig 1B). To avoid contamination, all steps of the preparation of PCR mixes were carried out in a laminar flow hood with aseptic techniques. In order to ensure no cross-reaction with fungi frequently found in cases involving CNS, *Cryptococcus neoformans* DNA was included as well (Fig 1B).

### Nested PCR

This reaction was performed according to the method described previously, to target the 18S rRNA gene [6], with slight modifications. Briefly, the reaction mixture of the first-round PCR consisted of 5 μl of DNA extract in a total volume of 50 μl, with final concentrations of 10 mM Tris-HCl (pH 9.0), 50 mM KCl, 0.1% Triton X-100, 1.5 mM MgCl_2_, 10μM concentrations of primers SS1 (5´-CTCGTTCGGCACCTTACACG-3´) and SS2 (5´-CGCTGCCAAAGCAACGCGGG-3´), 1.5 U of *Taq* polymerase (Invitrogen, USA), and a 200 μM concentration of each deoxynucleotide triphosphate (Invitrogen, USA). The reaction mixture of the nested PCR was identical, except that 3 μl of the first reaction product and the inner primer pair SS3 (5´-ACTCACCAGGTCCAGACACGATG-3´) and SS4 (5´-CGCGGGCTATTTAGCAGGTTAAG-3´) were used. Briefly, the PCR reaction was 95°C for 5 min and 40 cycles of 1 min at 95°C, 1 min at 68°C, and 1 min at 72°C, followed by 10 min at 72°C. PCR products were loaded onto agarose 2% w/v gels for electrophoresis and the gels stained with 0.5 mg per l of ethidium bromide. The first round amplified a 305 bp fragment and the second one, a 152bp fragment. The digital images were captured, and each experiment was repeated at least 3 times to ensure reproducibility.

## Results

Four patients were men, and one was woman, with ages from 25 to 44 years. All but one had neurological symptoms. All had CSF inflammatory parameters compatible with chronic meningoencephalitis, with no mass lesions. The cluster of differentiation 4 (CD4)+ T-cell count ranged from 11 mm^3^ to 302 mm^3^. Lumbar punctures were performed for all patients, with negative cultures in 4 cases for bacteria and fungi, and positive culture for *Sporothrix* spp. in only one case. Applying the mentioned nested PCR technique with the mentioned adjusts, we were able to detect the 152bp fragment from CSF of all the 5 patients tested, suggesting the presence of *Sporothrix sensu lato*, as previously described [6]. Negative control showed no amplification as well as positive controls for *C*. *neoformans*. In just one case, there was the isolation of *Sporothrix sensu lato*. No other agents were detected from the routine microbiological and immunological investigation.

## Discussion

CNS sporotrichosis is a challenge and is associated with a worsening of prognosis due to the difficult CSF sterilization [2]. Thus, it is pivotal a faster and more effective method for recognizing the fungus dissemination than mycological culture.

We are presenting an efficient approach for direct detection of *Sporothrix* DNA in specimens from sporotrichosis patients (Table 1). The paucity of pathogens, due to a low fungal burden in cases of CNS sporotrichosis, probably contributes to negative culture [4]. The nested PCR assay employed in our study provides a highly specific method to detect the *Sporothrix sensu lato* in CSF. It is important to highlight that the nested amplification of the 18S rRNA gene fragment can detect all *Sporothrix* species of the *Sporothrix* complex. Thus, our clinical sample had the presence of a *Sporothrix sensu lato*. The definition of the species depends on the development of new molecular strategies, which may be the aim for further studies.

**Table 1:** Advantages and disadvantages.

Advantages	Disadvantages
Fast diagnosis of sporotrichosis cases affecting CSF	Need laboratory structure for development of molecular technique
Early treatment start	Expensive DNA extraction kit
Avoid inappropriate treatment	Does not allow characterization at species level of the pathogen
High sensitivity and specificity to detect CSF sporotrichosis cases	Requires technical accuracy to perform the reaction

CSF, cerebrospinal fluid

This approach for a known technique is innovative and has the benefit to improve diagnosis and early treatment in patients with meningoencephalitis due to *Sporothrix sensu lato*.
